# Semidry acid hydrolysis of cellulose sustained by autoclaving for production of reducing sugars for bacterial biohydrogen generation from various cellulose feedstock

**DOI:** 10.7717/peerj.11244

**Published:** 2021-04-19

**Authors:** Fatthy Mohamed Morsy, Medhat Elbadry, Yasser Elbahloul

**Affiliations:** 1 Biology Department, Faculty of Science, Taibah University, Almadinah Almunawarah, Almadinah Almunawarah, Saudi Arabia; 2 Botany and Microbiology Department, Faculty of Science, Assiut University, Assiut, Assiut, Egypt; 3 Agricultural Microbiology Department, Faculty of Agriculture, Fayoum University, Fayoum, Fayoum, Egypt; 4 Botany and Microbiology Department, Faculty of Science, Alexandria University, Alexandria, Alexandria, Egypt

**Keywords:** Cellulose, Dark fermentation, *Escherichia coli*, Hydrogen gas, Polysaccharides, Semidry acid hydrolysis

## Abstract

Cellulosic biowastes are one of the cheapest and most abundant renewable organic materials on earth that can be, subsequent to hydrolysis, utilized as an organic carbon source for several fermentation biotechnologies. This study was devoted to explore a semidry acid hydrolysis of cellulose for decreasing the cost and ionic strength of the hydrolysate. For semidry acid hydrolysis, cellulose was just wetted with HCl (0 to 7 M) and subjected to autoclaving. The optimum molar concentration of HCl and period of autoclaving for semidry acid hydrolysis of cellulose were 6 M and 50 min respectively. Subsequent to the semidry acid hydrolysis with a minimum volume of 6 M HCl sustained by autoclaving, the hydrolysate was diluted with distilled water and neutralized with NaOH (0.5 M). The reducing sugars produced from the semidry acid hydrolysis of cellulose was further used for dark fermentation biohydrogen production by *Escherichia coli* as a representative of most hydrogen producing eubacteria which cannot utilize non-hydrolyzed cellulose. An isolated *E. coli* TFYM was used where this bacterium was morphologically and biochemically characterized and further identified by phylogenetic 16S rRNA encoding gene sequence analysis. The reducing sugars produced by semidry acid hydrolysis could be efficiently utilized by *E. coli* producing 0.4 mol H_2_ mol^−1^ hexose with a maximum rate of hydrogen gas production of 23.3 ml H_2_ h^−1^ L^−1^ and an estimated hydrogen yield of 20.5 (L H_2_ kg^−1^ dry biomass). The cheap cellulosic biowastes of wheat bran, sawdust and sugarcane bagasse could be hydrolyzed by semidry acid hydrolysis where the estimated hydrogen yield per kg of its dry biomass were 36, 18 and 32 (L H_2_ kg^−1^ dry biomass) respectively indicating a good feasibility of hydrogen production from reducing sugars prepared by semidry acid hydrolysis of these cellulosic biowastes. Semidry acid hydrolysis could also be effectively used for hydrolyzing non-cellulosic polysaccharides of dry cyanobacterial biomass. The described semidry acid hydrolysis of cellulosic biowastes in this study might be applicable not only for bacterial biohydrogen production but also for various hydrolyzed cellulose-based fermentation biotechnologies.

## Introduction

Cellulosic biowastes are of the most abundant renewable biomass available for hydrolysis for a cost-effective fermentation bioindustries. One of these fermentation biotechnologies is the production of hydrogen gas by bacteria. Hydrogen gas is one of the applicable sources of future renewable energy that is potentially promising to replace the present worldwide used fossil fuels (Yokoi et al., 2001; Nath & Das, 2004; Kapdan & Kargi, 2006; Chong et al., 2009; Abd-Alla, Morsy & El-Enany, 2011; Budiman & Wu, 2018; Noblecourt et al., 2018; Morsy et al., 2019). The hydrogen gas production by eubacteria is a cost-effective way where cheap organic wastes can be utilized in such future biological industry  (Yu et al., 2002; Zhang, Brunsb & Logan, 2006; Pattra et al., 2008; Lo et al., 2009; Cheng & Chang, 2011; Noparat, Prasertsan & Sompong, 2011; Hay et al., 2016; Morsy, 2017; Morsy, Elbahloul & Elbadry, 2019). Cellulose feedstock come at the top of interest to be utilized as an organic form source of carbon for producing H_2_ by eubacteria, however most hydrogen producing eubacteria cannot utilize cellulosic biowastes. Hydrolysis of cellulose is thus fundamental for an efficient utilization of cellulosic biowastes in the production of hydrogen by many eubacteria. The efficiency of acid hydrolysis of polysaccharides in general depends on its polymeric complexity where cellulose is one of the difficult types of polysaccharides to hydrolyze. Due to abundant cellulose feedstock all over the world (Sukumaran, Singhania & Pandey, 2005), its hydrolysis is of the hot topics for the production of reducing sugars for various biofuels fermentation biotechnological industries. In the past, cellulosic feedstocks were used in heating but now with the modernization of developing countries, it is no longer used and replaced by biogas. Thus, farmers in developing countries sometimes burn most of the crop plants straw in-situ of their farms to get rid of it. This leads to a high-risk pollution and increases the CO_2_ in the atmosphere; a trouble that shares to some extent, besides CO_2_-liberating industries and extensive use of fossil fuels, in increasing the percent of CO_2_ gas in the atmosphere enhancing global warming. Many works of literature seek useful utilization of cellulose feedstocks in producing hydrogen gas biologically. Acid and enzymatic hydrolysis of the polysaccharide cellulose is usually used for producing reducing sugars (Camacho et al., 1996; Iranmahboob, Nadim & Monemi, 2002; Sun & Cheng, 2002; Xiang et al., 2003; Taherdazeh & Karimi, 2007; El-Zawawy et al., 2011; Heinonen et al., 2012; Ni et al., 2013; Pulidindi, Kimchi & Gedanken, 2014; Vo et al., 2014; Yoon, Han & Shin, 2014). The acid hydrolysis is more applicable in high mass of cellulose at industrial scale. Reducing the amount of acid used in the hydrolysis is useful in two aspects; first it reduces the cost by reducing the amount of acid per-se and the amount of base used in subsequent neutralization step; second it will reduce the final ionic strength of the fermentation medium that might adversely affect the fermentation process. In this study a semidry acid hydrolysis of cellulose was conducted and its efficiency in producing reducing sugars was investigated. Applicability of acid hydrolysis of lignocelluloses and other polymeric carbohydrates is extensively reported in many fields of biotechnologies (Cuzens & Miller, 1997; Baruah et al., 2018; Bhatia et al., 2021). Hydrogen gas production by *E. coli* strain TFYM from hydrolyzed pure cellulose and various cellulose feedstock was investigated as an example of the many biotechnological applications that can be served by semidry acid hydrolysis of cellulose in a cost-effective way.

## Materials and Methods

### Semidry acid hydrolysis of cellulose

Cellulose [Cellulose powder RM126, HiMedia Laboratories Pvt. Ltd. India], retaining no humidity confirmed by drying at 70 °C for 20 h and re-weighting and stored in dry conditions, was used for semidry acid hydrolysis where it was just wetted by HCl in a ratio of 1:1 [1g cellulose: 1 ml HCl], in loosely screw capped glass tubes, followed by autoclaving at 121 °C using LabTech vertical autoclave (Model LAC-51005, Daihan LabTech Co., LTD, Namyangju-City, Kyonggi-Do, Korea).

#### Optimization of HCl molar concentration and period of autoclaving for semidry acid hydrolysis of cellulose

Optimization experiments of semidry acid hydrolysis was conducted in loosely screw capped glass tubes retaining 1 g cellulose and 1 ml of 0 (H_2_O) to 7 M HCl (255.2 g/L). For optimization of HCl molar concentration and period of autoclaving for semidry acid hydrolysis of cellulose, optimum molarity of HCl for hydrolysis was determined first. The optimization of HCl molar concentration for semidry acid hydrolysis of cellulose was conducted using various molarities of HCl (0 to 7 M) at a constant period of autoclaving for 30 min. Subsequently, the optimization of the autoclaving period for semidry acid hydrolysis of cellulose was investigated at constant optimum molarity of HCl using various autoclaving periods (0 to 70 min). The determined optimum molarity was 6 M HCl for semidry acid hydrolysis of cellulose and thus, determination of the optimum period of autoclaving was conducted at constant 6M HCl. The period (0.0) of autoclaving is after acid treatment (addition of 6M HCl to cellulose) before autoclaving. Control samples with no acid treatment (acid replaced by distilled water) were subjected to the various periods of autoclaving used.

#### Hydrolysate neutralization and preparation

Subsequent to autoclaving, the hydrolysate was diluted with distilled H_2_O and filtered through Whatman No. 1 filter paper (Cat. No. 1001-090, Whatman International Ltd, Maidstone England). The hydrolysate filtrate was neutralized by 0.5 M NaOH solution and the reducing sugars content was measured by Nelson reagent (Nelson, 1944).

#### Semidry acid hydrolysis of various cellulosic biowastes

Semidry acid hydrolysis was applied on various cellulose feedstocks including wheat bran, sawdust and sugarcane bagasse. Wheat bran was purchased from the market. Sawdust was obtained from a carpenter shop. Sugarcane bagasse was obtained from Egypt and ground to small pieces. All feedstocks were washed with distilled water, filtered through cloth filter and dried at 70 °C up to constant weight, stored in dry conditions and subsequently used for semidry acid hydrolysis. Cellulose and various cellulose feedstocks (wheat bran, sawdust and sugarcane bagasse) were subjected to semidry acid hydrolysis in loosely screw capped glass bottles (250 ml SCHOTT Duran-group, Germany) retaining 10 g dry biomass and 10 ml of optimum HCl molar concentration (6M HCl) in a ratio of 1:1 [1g biomass: 1 ml HCl] and subjected to optimum autoclaving period (50 min.). The hydrolysates were diluted, filtered and neutralized as described above. The released reducing sugars content in the filtrates was measured by Nelson reagent (Nelson, 1944) where the efficiency of the semidry acid hydrolysis represented in the percent hydrolysis of the cellulosic biowastes was calculated as follows:

\begin{eqnarray*}\text{% Hydrolysis}=[\text{Amount of reducing sugars released}(\text{g})/\text{Dry mass}(\text{g})]\times 100 \end{eqnarray*}

The neutralized filtrate retaining the released reducing sugars from semidry acid hydrolysis of cellulose and various cellulosic feedstocks were used subsequently for dark fermentation hydrogen gas production by *E. coli* TFYM.

### Isolation and identification of *E. coli* TFYM

Isolation of *E. coli* and its phenotypic characterization was conducted by standard protocols. Isolation of the bacterium *E. coli* TFYM was conducted using lactose broth following the techniques [MPN (Most Probable Number)] (MacFaddin, 1985) from wastewater sample at Saudi Arabia. Confirmation of the positive tubes was performed using Eosin Methylene Blue (EMB) agar and it was further characterized as *E. coli* bacterium on MacConkey medium. Other characterizations of the bacterium were conducted following Bergey’s Manual (Brenner et al., 2005).

### Molecular biological identification and phylogenetic analysis of *E. coli* TFYM 16S rRNA gene sequence

#### The 16S rRNA gene amplification by PCR

The bacterial cells genomic DNA was extracted by Promega Wizard Genomic DNA Purification Kits (Promega, USA) according to the kit manufacturer instructions. Subsequently, the 16S rRNA encoding gene amplification was conducted to a near-full length by PCR using the genomic DNA template and the universal forward 27F primer with a sequence of (5′-AGAGTTTGATC[A/C]TGGCTCAG-3′) and reverse 1492R universal primer with a sequence of (5′-G[C/T]TACCTTGTTACGACTT-3′) (Lane, 1991). The amplification by PCR was performed in a reaction mixture (25 µl) composed of 2.5 µl of 10 × *Taq* buffer (100 mM Tris–HCl, pH 8), 100 µM dNTPs, 1.25 mM MgCl_2_, 1.2 µM forward and reverse universal primers, 0.5U of the *Taq* DNA polymerase, in addition to the genomic DNA of about 5 ng as a template. The PCR amplification was performed using Model 2720, USA Applied Biosystem Thermal Cycler following a program for PCR of 5 min at 95 °C (initial denaturation), 35 cycles of [1 min at 94 °C (denaturation), 1 min at 56 °C (annealing), and 1 min at 72 °C (extension)] followed by 10 min at 72 °C as a final extension. The PCR amplification product was subsequently analyzed by standard protocol of agarose-electrophoresis using 1% agarose-gel retaining 5 µg/mL of ethidium bromide. A 1 kb Plus DNA ladder size marker (Invitrogen, USA) was used.

#### Sequencing, accession number and phylogenetic analysis of 16S rRNA encoding gene nucleotides sequence

The amplification product of the PCR was purified by PCR Purification Kit [Invitrogen PureLink (Invitrogen, USA)]. Subsequently the purified PCR product was photometrically quantified. Using same forward and reverse primers, the PCR purified product was cycle sequenced in both directions using automated florescent dye terminator sequencing method (Sanger, Nicklen & Coulson, 1977) at Macrogen Korea sequencing facility, (Seoul, Korea). The 16S rRNA encoding gene sequence reads were assembled and compared with its nearest matches found by searching in the nucleotide-nucleotide BLAST of the GenBank website search tools of the NCBI website. The alignments of the base sequences of the 16S rRNA encoding gene were conducted using the website of Clustal W1.83 XP  (Thompson et al., 1997). The 16S rRNA gene sequence derived phylogenetic tree was constructed through the use of neighbor-joining method (Saitou & Nei, 1987) using MEGAX software (Kumar et al., 2018). *Bacillus cereus* strain ATCC14579 (NR_074540.1) was used as outgroup. The obtained base sequence in this study of *E. coli* TFYM 16S rRNA encoding gene has been deposited as near full length sequence of this gene in the GenBank website of nucleotide sequence database under accession number MK332445.1.

### Growth of *E. coli* on reducing sugars prepared by semidry acid hydrolysis of cellulose as indicator of its utilization of as a carbon source

The utilization of semidry acid hydrolyzed cellulose by *E. coli* for growth was investigated using BM (Basal Mineral) medium supplemented with 5 g/L reducing sugars of semidry acid hydrolyzed cellulose. BM medium was composed of the followings (per liter): K_2_HPO_4_, 4.4 g; (NH_4_)_2_SO_4_, 1.3 g; NaH_2_PO_4_, 3.5 g; MgSO4.6H_2_O, 0.9 g and 1 ml of trace elements solution. The trace elements solution was composed of the followings (per 100 ml): FeSO_4_ .7H_2_O, 0.37 g; CaCl_2_.2H_2_O, 4.8 g; MnCl_2_, 0.1 g; CoCl_2_.6H_2_O, 0.04 g; Na_2_MoO_4_.2H_2_O, 0.02 g. The aerobic growth of *E. coli* on reducing sugars of semidry acid hydrolysis of cellulose on basal medium (BM) was followed spectrophotometrically by absorbance at wavelength 600 nm quantified using UV/Vis spectrophotometer (6320D Jenway). A calibration of the growth followed by OD at 600 nm versus dry cell weight (DCW) was conducted in 100 ml cultures of *E. coli* on reducing sugars of semidry acid hydrolysis of cellulose in Basal Mineral (BM) medium.

### Hydrogen gas production by *E. coli* TFYM dark fermentation

Prior to fermentation, *E. coli* TFYM was grown in LB medium [10 g/l tryptone, 10 g/l NaCl, 5 g/l yeast extract] at 35 °C. *E. coli* TFYM batch dark fermentation experiments were conducted for H_2_ formation from reducing sugars prepared as described above by semidry acid hydrolysis of cellulose and cellulosic feedstock biowastes (wheat bran sugarcane bagasse and sawdust). A one-liter glass fermentation bottle was used with a working volume of 970 ml. A volume 870 ml of neutralized hydrolysate was put in the fermentation bottle and supplemented with *E. coli* TFYM (OD_600_ equal 0.25) 100 ml inoculum. The fermentation bottle was closed with a rubber bung and subsequently sparged with nitrogen gas for 20 min to accelerate installing anaerobic conditions. The fermentation bottle was kept in dark with continuous stirring at 35 °C. The evolved H_2_ gas was collected using a CO_2_-free H_2_ gas collection system (Morsy, 2015) relying on passing the evolved gas on NaOH solution for absorbing CO_2_ and subsequently collecting the CO_2_-free H_2_ gas by water displacement. The collected H_2_gas compared to pure H_2_ gas was measured using a Clark-type platinum-coated electrode computerized system [purchased from (Hansatech Instruments, Inc.) for Taibah University, Saudi Arabia] according to manufacturer instructions. The rate of H_2_production was estimated as previously described (Morsy, Elbahloul & Elbadry, 2019) at each point of measurements of the produced cumulative H_2_ gas as follows:

\begin{eqnarray*}\text{Rate of} {\mathrm{H}}_{2} \text{gas production}=({V}_{\mathrm{x}}-{V}_{\mathrm{p}})/({T}_{\mathrm{x}}-{T}_{\mathrm{p}}) \end{eqnarray*}

Where (*V*_x_ − *V*_p_) is the difference in volume of collected cumulative H_2_ gas at measurement time point (x) and the previous measurement time point (p). (*T*_x_ − *T*_p_) is difference in time between the two cumulative H_2_gas measurement points.

**Figure 1:**
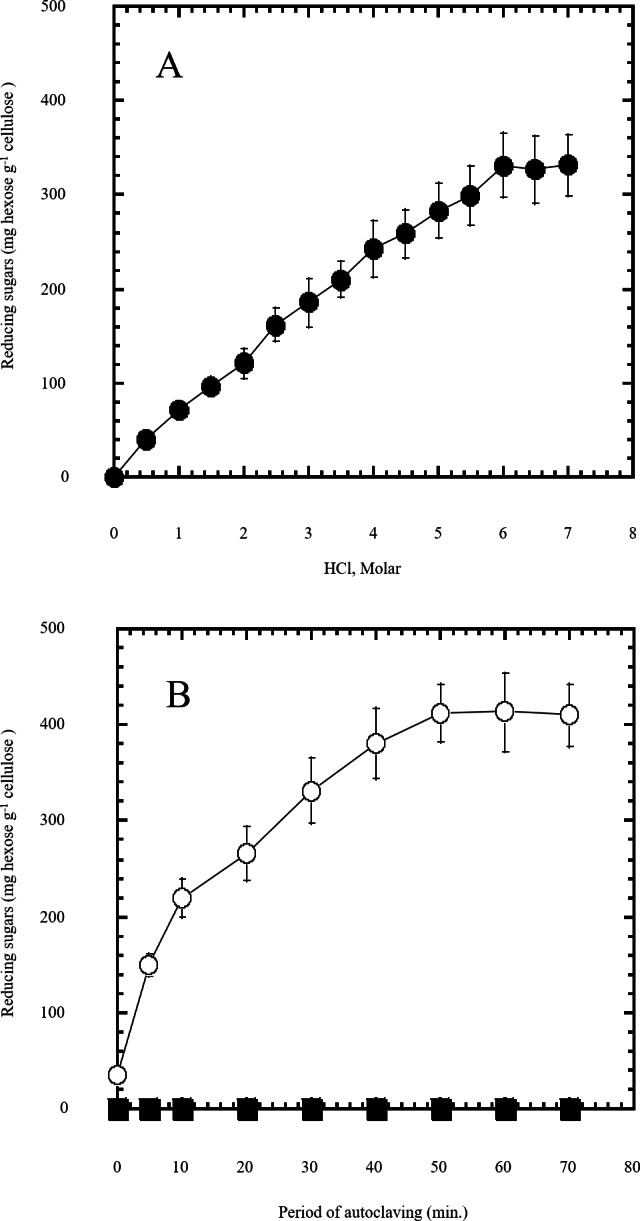
Semidry acid hydrolysis of cellulose. (A) shows the optimization of HCl molar concentration in semidry acid hydrolysis of cellulose sustained by autoclaving for 30 min. Semidry acid hydrolysis of cellulose using various molarities of HCl for a constant period of autoclaving for 30 min. (B) shows the autoclaving period optimization for semidry acid hydrolysis of cellulose at 6M HCl. The determined optimum molarity of 6 M HCl for semidry acid hydrolysis of cellulose was used (open circles) for determining the optimum period of autoclaving. Control samples (closed squares) with no acid treatment (replaced by water) were subjected to various periods of autoclaving used. The mean values of three replicates and standard errors are shown.

## Results

**Figure 2:**
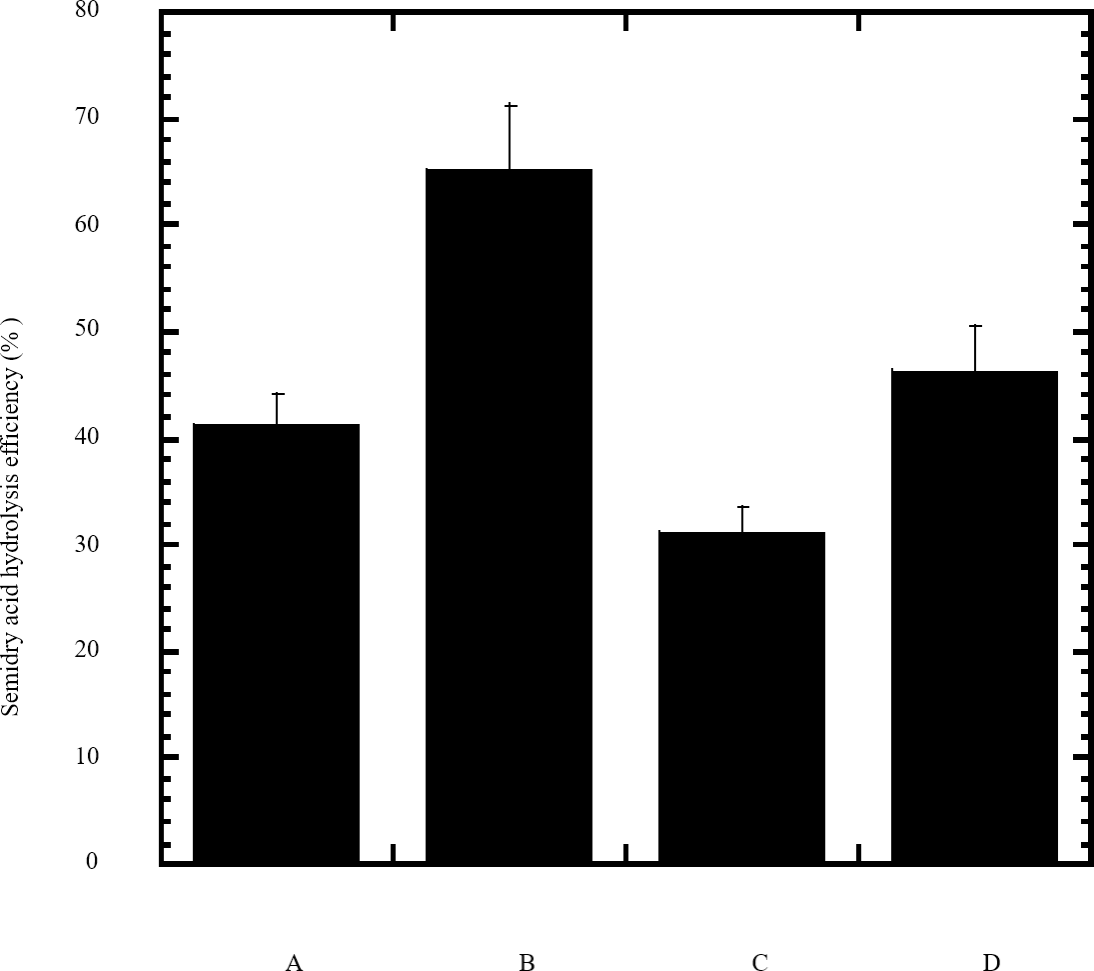
Efficiency of semidry acid hydrolysis of cellulose. The efficiency of semidry acid hydrolysis of various cellulosic feedstock are represented in percent hydrolysis of pure cellulose (column A), wheat bran (column B), sawdust (column C), sugarcane bagasse (column D). The mean values of three replicates and standard errors are shown.

**Figure 3:**
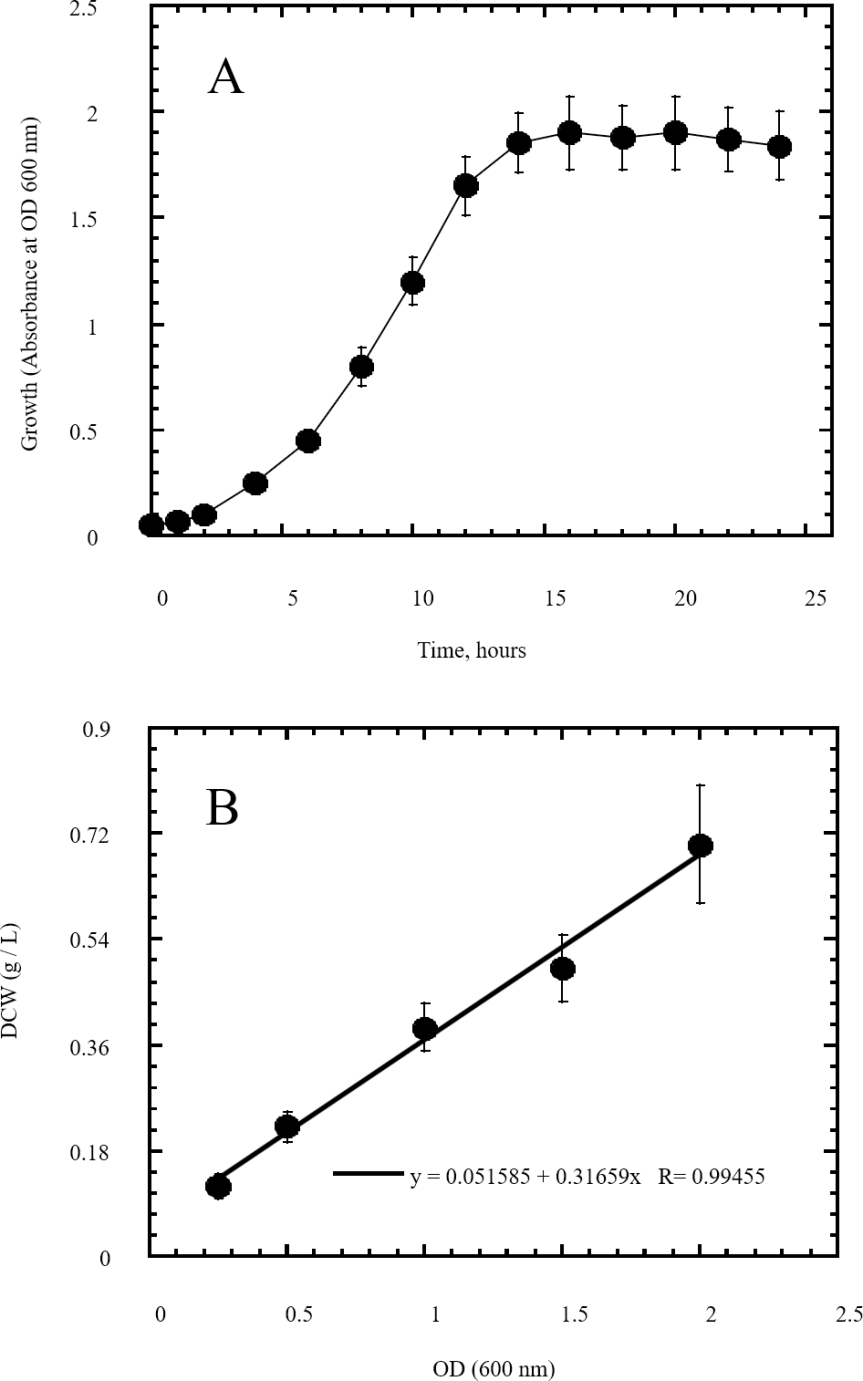
Growth of *Escherichia coli* as indicator of its utilization of reducing sugars prepared by semidry acid hydrolysis of cellulose as a carbon source. The growth of *Escherichia coli* was conducted on basal mineral medium supplemented with hydrolysate of cellulose and followed photometrically at 600 nm (A) to explore the ability of *Escherichia coli* for utilizing the reducing sugars produced from semidry acid hydrolysis of cellulose. (B) shows the calibration of OD (600 nm) versus dry cell weight (DCW) of *Escherichia coli* aerobic growth on reducing sugars of semidry acid hydrolyzed cellulose in Basal Mineral (BM) medium. The mean values of three replicates and standard errors are shown.

**Figure 4:**
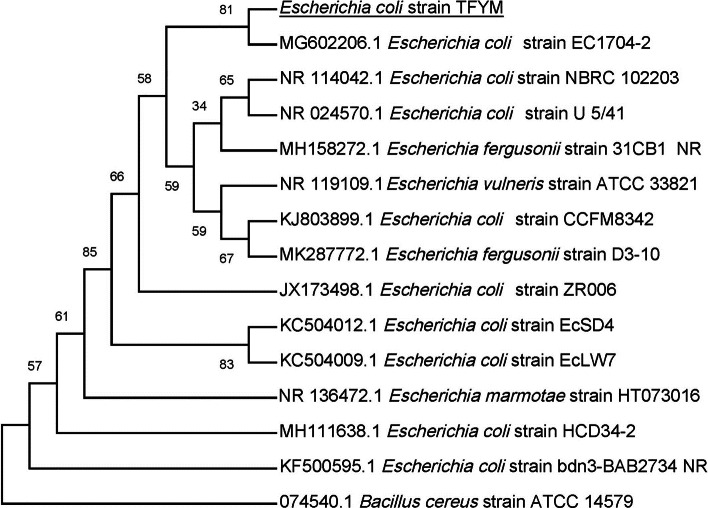
Phylogenetic tree of isolated *Escherichia coli* TFYM indicating the relationship of this strain with its nearest bacterial strains neighbors from NCBI. The evolutionary relationships of *Escherichia* sp. TFYM to other species of *Escherichia* sp were deduced using the Neighbor-Joining method to represent the taxa analyzed evolutionary history (Felsenstein, 1985). The evolutionary comparisons considered the variations in the composition bias among sequences (Tamura & Kumar, 2002). Bacillus cereus strain ATCC14759 was used as outgroup for comparison. The evolutionary analysis were performed using MEGA X.

**Figure 5:**
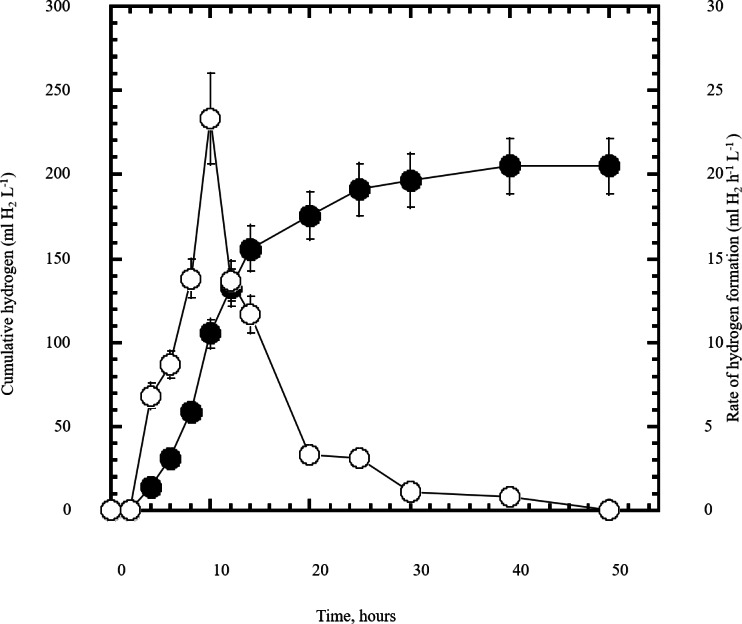
Dark fermentation cumulative hydrogen gas production by *Escherichia coli* TFYM from reducing sugars prepared by semidry acid hydrolysis of cellulose. The cumulative hydrogen gas production (closed circles) by *Escherichia coli* TFYM dark fermentation was followed. The rate of hydrogen gas production (open circles) was estimated along the fermentation period. The mean values and standard errors of three independent fermentation experiments are shown.

**Figure 6:**
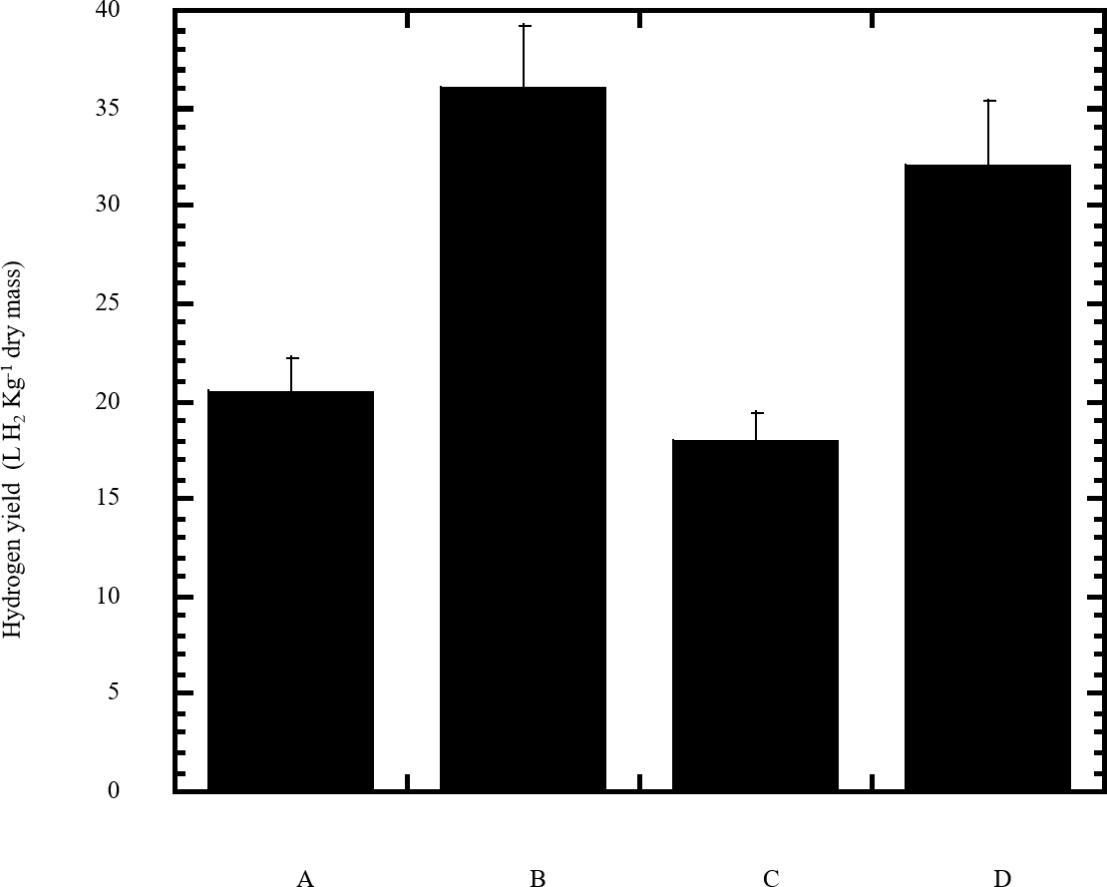
Estimated hydrogen gas yield of *Escherichia coli* TFYM from reducing sugars prepared by semidry acid hydrolysis of various cellulosic feedstock. The hydrogen yield was estimated for dark fermentation by Escherichia coli TFYM from reducing sugars of semidry acid hydrolyzed cellulose (column A), wheat bran (column B), sawdust (column C) and sugarcane bagasse (column D). The mean values and standard errors of three independent fermentation experiments are shown.

In this study a semidry acid hydrolysis of cellulose and cellulosic feedstock biowastes was conducted for producing reducing sugars that can be used in many fermentation biotechnologies including production of hydrogen gas by bacterial dark fermentation. The semidry acid hydrolysis of cellulose was performed in a ratio of 1:1 [1 g biomass:1 ml of HCl where the cellulose was just wetted by the acid and subjected to autoclaving. The optimum HCl molar concentration for semidry acid hydrolysis of cellulose was 6 M determined first at autoclaving period of 30 min (Fig. 1A). The optimum autoclaving period was subsequently determined as 50 min (Fig. 1B) at optimum 6M HCl. These optimum 6M HCl and 50 min autoclaving conditions was efficient (Fig. 2) in hydrolyzing various cheap cellulosic feedstock biowastes (wheat bran, sawdust and sugarcane bagasse). The percent hydrolysis of wheat bran [65% ± 10.58 (Standard Deviation)] and sugarcane bagasse (46.3% ± 7.18) was more than pure cellulose (41.2% ± 5.31) possibly due to hydrolyzing cellulose and other less complicated more hydrolysable polysaccharides such as hemicellulose and others that is known to be present in natural plant residues. The semidry acid hydrolysis was also applied on non-cellulosic biomass of the potent extracellular polysaccharides producing cyanobacterium *Nostoc commune* where it produced 0.51 ± 0.12 g reducing sugars/ g dry mass. The ability of *E. coli* to utilize the reducing sugars produced by semidry acid hydrolysis of cellulose was conducted on Basal Mineral (BM) medium, so that the medium contains only minerals and a soli organic carbon source (the reducing sugars of semidry acid hydrolyzed cellulose). The results showed good ability of *E. coli* for utilization of the reducing sugars prepared by semidry acid hydrolysis of cellulose as investigated in a basal mineral medium where the bacterium could grow efficiently (Fig. 3) using the hydrolysate reducing sugars as an organic carbon source. The aerobic growth of *E. coli* on reducing sugars of semidry acid hydrolysis of cellulose in Basal Mineral (BM) medium followed by OD at 600 nm (Fig. 3A) was calibrated (Fig. 3B) versus dry cell weight (DCW) where a calibration value of OD_600 nm_ of 1 equals 0.368 g/L. *E. coli* like most other hydrogen producing bacteria cannot utilize cellulose as a carbon source and its utilization of reducing sugars prepared by semidry acid hydrolysis is a representative for efficiency of using such hydrolysate in dark fermentation. The bacterium used in this study was *E. coli* TFYM as a representative of the hydrogen producers to investigate the suitability of the hydrolysate prepared by semidry acid hydrolysis. This isolated bacterium was identified morphologically and biochemically (Table 1) following standard protocols. The identification was also confirmed by phylogenetic analysis of the 16S rRNA encoding gene sequence (Fig. 4). As the bacterium could utilize the reducing sugars in the hydrolysate prepared by semidry acid hydrolysis of cellulose, it was used for investigating the suitability of the hydrolysate for dark fermentation biohydrogen gas production. For the batch dark fermentation hydrogen production, the semidry acid hydrolysis of 10 g cellulose or various cellulose feedstock of wheat bran, sawdust and sugarcane bagasse was conducted and the hydrolysate retaining the reducing sugars was filtrated and neutralized to be used as an organic source of carbon for hydrogen formation by *E. coli* TFYM dark fermentation. The rate of hydrogen gas production gradually increased and was maximum (23.3 ml H_2_ h^−1^ L^−1^) at 10 h after the start of fermentation followed by a decline at the late stationary phase of cumulative hydrogen production (Fig. 5). The reducing sugars produced by semidry acid hydrolysis (4.12 g/ 10 g cellulose) were efficiently used for hydrogen production by *E. coli* producing 0.4 mol H_2_ mol^−1^ hexose which is comparable to previously reported yield by *E. coli* from expensive pure sugars and sugar wastes (Table 2). The estimated hydrogen yield by *E. coli* (Fig. 6) from the reducing sugars prepared by the semidry acid hydrolysis of the cheap cellulosic biowastes of wheat bran, sawdust and sugarcane bagasse was 36, 18 and 32 (L H_2_ kg^−1^ dry biomass) respectively. These results indicate a good feasibility of hydrogen production from reducing sugars prepared by semidry acid hydrolysis of such cheap cellulosic biowastes.

**Table 1:** Morphological and biochemical identification characteristics of *E. coli* strain TFYM.

**Characteristics**	***Escherichia coli***	**Strain TFYM**
Morphological features		
Colonies on EMB agar	Green Metallic sheen	Green Metallic sheen
Gram staining of cell wall	-ve	-ve
3% KOH	Viscous and thread like slime	Viscous and thread like slime
Cell shape	Unicellular short rods	Unicellular short rods
Bacterial cell motility	Motile	Motile
Spore	-ve	-ve
Biochemical tests		
Characteristic Growth on MacConkey	+ve	+ve
Catalase test	+ve	+ve
Oxidase test	-ve	-ve
Methyl Red (MR)	+ve	+ve
Indole	+ve	+ve
Voges-Proskauer (VP)	-ve	-ve
Citrate	-ve	-ve
Gas	+ve	+ve
Presumptive test	+ve	+ve
Urease	-ve	-ve
Gelatin liquefaction	-ve	-ve

**Table 2:** Hydrogen yield by *E. coli* from semidry acid hydrolyzed pure cellulose in comparison to other non-cellulosic carbohydrates.

**Microorganism(s)**	**substrate**	**Yield [mol of H**_**2**_**/ molof hexose]**	**Ref.**
*Escherichia coli* HD701	Acid hydrolyzed potato starch residue stream	0.45	Morsy (2014)
*Escherichia coli* W3110	Glucose	0.54	Fan, Yuan & Chatterjee (2009)
*Escherichia coli* ZF3^a^	Glucose	0.96	Fan, Yuan & Chatterjee (2009)
*Escherichia coli S3*	Glucose	0.84	Junyapoon, Buala & Phunpruch (2011)
*Escherichia coli* SH3^b^	Glucose	1.48	Kim et al. (2009)
*Escherichia coli*^c^	Glucose	**0.17**	Manish, Venkatesh & Banerjee (2007)
*Escherichia coli Δ ldh*^d^	Glucose	**0.23**	Manish, Venkatesh & Banerjee (2007)
*Escherichia coli* HD701	Acid hydrolyzed molasses	**0.46**	Morsy (2011)
*Escherichia coli* DJT135^e^	Glucose	**1.51**	Ghosh & Hallenbeck (2009a)
*Escherichia coli* DJT135	Fructose	**1.27**	Ghosh & Hallenbeck (2009a)
*Escherichia coli* DJT135	Galactose	**0.69**	Ghosh & Hallenbeck (2009a)
*Escherichia coli* DJT135	Glucose	**1.69**	Ghosh & Hallenbeck (2009b)
*Escherichia coli* BW25113^f^	Glucose	**1.35**	Maeda, Sanchez-Torres & Wood (2007)
*Escherichia coli* SR15^g^	Glucose	**1.82**	Yoshida et al. (2006)
*Escherichia coli* WDHL^h^	Galactose	**1.12**	Rosales-Colunga, Razo-Flores & Rodriguez (2012)
*Escherichia coli* WDHL	Lactose + galactose	**1.02**	Rosales-Colunga, Razo-Flores & Rodriguez (2012)
*Escherichia coli* WDHL	Glucose + galactose	**1.02**	Rosales-Colunga, Razo-Flores & Rodriguez (2012)
*Escherichia coli* WDHL	Glucose	**0.3**	Rosales-Colunga, Razo-Flores & Rodriguez (2012)
*Escherichia coli* strain TFYM	Semidry acid hydrolyzed pure cellulose	0.4	This study

**Notes.**

a *Escherichia coli* ZF3 mutant strain (Deletion of *narL*)

b *Escherichia coli* strain SH3 mutant engineered from disrupting the genes encoding two uptake hydrogenases; hydrogenase 1 (*hya*) and hydrogenase 2 (*hyb*)

c *Escherichia coli* (The parent strain for *Escherichia coli* Δ *ldh*)

d *Escherichia coli* Δ *ldh* mutant strain that lacks the enzyme lactate dehydrogenase

e *Escherichia coli* DJT135 mutant strain (Deletion of uptake hydrogenases, mutation of *ldhA* and constitutive expression of *fhl*)

f *Escherichia coli* BW25113 mutant strain (Deletion of *hyaB*, *hybC*, *hycA*, *fdoG*, *frdC*, *ldha* and *aceE*)

g *Escherichia coli* SR15 mutant strain (Deletion of *ldhA* and *frdBc*)

h *Escherichia coli* WDHL mutant strain (Deletion of *lacI* and *hycA*)

## Discussion

Cellulosic biomass wastes are cheap and abundant. Thus, upon hydrolysis, these polymeric renewable organic materials can be used in many fermentation biotechnological industries. This study describes a semidry acid hydrolysis of cellulosic feedstock biowastes where the biomass was just wetted with 6M HCl and subjected to autoclaving for 50 min where a considerable amount of reducing sugars were produced where the percent hydrolysis of pure cellulose was 41.2%. Assessment of semidry acid hydrolysis of cellulose was conducted using pure cellulose in first part of the study where 412 ± 53.1 mg reducing sugars/g of pure cellulose were produced. Subsequently application of semidry acid hydrolysis on crude cellulosic biowastes of wheat bran, sawdust and sugarcane bagasse biomasses was conducted. The cellulose, hemicellulose, lignin and other organic materials composition of these crude biowastes were previously reported (Table 3) where cellulose is basic structural components of these biowastes. In comparison to pure cellulose, semidry acid hydrolysis showed higher percent of hydrolysis in case of wheat bran (65%) and sugarcane bagasse (46.3%) mostly due to presence of complicated more hydrolysable polysaccharides associated with cellulose in these natural plant residues such as hemicellulose which is present along with cellulose in wheat bran (Merali et al., 2015), sugarcane bagasse  (Sanjuán et al., 2001) and almost all terrestrial plant cell walls (Scheller & Ulvskov, 2010). The ground tissue in sugarcane stalks retains abundant parenchyma cells whose cell wall is primary one which is composed mainly of cellulose and hemicellulose. Subsequent to crushing sugarcane stalks for extracting its juice by crusher machine in the initial steps of sugar industry, the sugarcane bagasse obtained is rich in residues of the primary cell walls of parenchyma tissue retaining cellulose and hemicellulose. Thus, these natural waste biomasses, including sugarcane bagasse and also wheat bran retaining both cellulose and the less complicated hemicellulose, are highly susceptible for semidry acid hydrolysis. This indicates that semidry acid hydrolysis is efficient for hydrolyzing cellulose and other less complicated polymeric carbohydrates in the cellulosic feedstock wastes. The semidry acid hydrolysis described in this study was thus highly efficient for hydrolysis of non-cellulosic cyanobacterial biomass. The potent extracellular polysaccharides producing (Hill, Peat & Potts, 1994; Hill et al., 1997; Helm et al., 2000; Tamaru et al., 2005; Morsy et al., 2008) cyanobacterium *Nostoc commune* biomass was efficiently hydrolyzed (51%) by semidry acid hydrolysis indicating that semidry acid hydrolysis was highly effective for hydrolyzing also this less complicated non-cellulosic renewable biomass. Subjecting cellulosic biomaterials to high concentrated H_2_SO_4_ in a two-step hydrolysis was described elsewhere (Iranmahboob, Nadim & Monemi, 2002; Chang et al., 2018). A two step acid hydrolysis was described to hydrolyze wood chips where the first step included treatment with high concentrated 80% H_2_SO_4_ at room temperature with a mass ratio of 500 g H_2_SO_4_/200 g dry mass of wood chips followed by addition of distilled boiling water to reach 26 wt% H_2_SO_4_ with boiling for 30 min and stirring followed by filtration where the filtrate containing cellulose was heated in a second step for extra 2 h resulting in 78–82% overall hydrolysis efficiency of cellulose theoretical values in wood (Iranmahboob, Nadim & Monemi, 2002). The Two-Step cellulose hydrolysis was also described elsewhere where in the first step cellulose was treated with high concentrated 72 wt% H_2_SO_4_ at 30 °C over 2 h in a ratio of H_2_SO_4_/dry mass of cellulose of 36 followed by a partial second step neutralization through using 20 wt% NaOH at 2.3–2.5 molar ratio for H^+^/OH^−^ with subjecting to more hydrolysis 10 min autoclaving at a temperature of 121 °C (Chang et al., 2018). Cotton cellulose was found to completely dissolve at room temperature in high concentrated sulfuric acid above 55% (by volume) and a reduced sugar yields from the initial cotton cellulose concentrations of 30–70 g/L were varied from 64.3 to 73.9% (g R-sugar/g cotton cellulose) at a temperature of 40 (Chu et al., 2011). The percent hydrolysis of sawdust was lower than cellulose possibly due to presence of highly complicated lignin in wood and lower cellulose content. Around 20% to 30% of sawdust content is lignin (Sınağ et al., 2009) which is highly complicated to be hydrolyzed. However, the use of sawdust for obtaining reducing sugars through semidry hydrolysis would depend in the source of the sawdust where the cellulose content would depend on the type of wood. The resulted reducing sugars were utilizable by *E. coli* as a representative of the fermenting bacteria indicating that the reducing sugars prepared by semidry acid hydrolysis are fermentable and can be utilized not only for dark fermentation hydrogen production but also for other fermentation-based biotechnologies. In this study, the organic carbon sources (the reducing sugars of semidry acid hydrolyzed cellulose and various cellulosic feedstocks) used for dark fermentation hydrogen production, are already subjected to autoclaving during semidry acid hydrolysis and hence it contains no native microbiota. Thus, the effect of native microbiota described elsewhere (Dauptain et al., 2020) is not applicable in the present study where fermentation was conducted by the supplied *E. coli* inoculum. Confirmative control experiments with no *E. coli* inoculated to the fermentor, did not produce hydrogen gas indicating no native microbiota effect is there where *E. coli* is the soli hydrogen producing bacterium in the fermentor through its utilization of the reducing sugars of semidry acid hydrolyzed cellulose and various cellulosic feedstocks. *E. coli* produce only hydrogen and CO_2_ gas (Penfold, Forster & Macaskie, 2003) where CO_2_ is absorbed by NaOH in the collection system and the collected hydrogen gas was fully pure compared to reference samples of pure hydrogen in measurements. The use of the abundant cellulose feedstock for hydrogen production would be cost effective through the semidry acid hydrolysis. The feasibility of such industry would be of importance in future upon exhaustion of fossil fuels (Muradov & Veziroglu, 2008). In fact, more and more research on the valorization of agricultural residual into biofuel has attracted great attention, mainly due to the positive effects from both economic and environmental aspects and long-term energy sustainability with greenhouse gas mitigation (Ho, Ong & Wu, 2019). The attempt to use cellulose feedstock would also be of importance in many agricultural countries. Besides, the re-activation of this biological hydrogen production industry would encourage farmers in developing countries to make use of crop plants straw and avoiding the harsh burning of such straw and cellulosic agricultural wastes. The data shown in this study either for growth of *E. coli* on the reducing sugars obtained from semidry acid hydrolysis or its utilization for hydrogen production did not show any inhibition or toxicity against the bacterium by the hydrolysate contents. As it requires minimum amount of acid and hence minimum amount of base for subsequent neutralization step, the semidry acid hydrolysis of cellulosic biowastes would be cost effective for bacterial hydrogen production biotechnology. The semidry acid hydrolysis of the cheap and abundant cellulosic wastes feedstock might possibly be applicable not only for bacterial H_2_ production but also for other cellulose dependent biotechnologies. The described semidry acid hydrolysis reduces the amount of high molarity HCl required for hydrolysis to minimum. Thus, the amount of NaOH required for the tedious neutralization step required for various fermentation biotechnologies comes to minimum. Further future studies for modification of semidry acid hydrolysis such as combination with other hydrolysis protocols would be of interest for the best making use of the abundant cellulosic biowastes in various fermentation biotechnologies.

**Table 3:** Cellulose, hemicellulose and lignin contents of wheat bran, sawdust and sugarcane bagasse biomasses.

**Biomass**	**Cellulose**	**Hemicellulose**	**Lignin**	**References**
Sawdust	40–50%	25–35%	20–30%	Sınağ et al. (2009)
Wheat bran	31.4 ± 1.6%	20.3 ± 1.0%	22.3 ± 0.3%	Cantero et al. (2015)
Sugarcane bagasse	32–45%	20–32%	17–32%	Alokikaa et al. (2021)

##  Supplemental Information

10.7717/peerj.11244/supp-1 Data S1 Raw data

## Additional Information and Declarations

### Data Availability

The following information was supplied regarding data availability: Raw data for Figures 1, 2, 3, 4, 6, and 7 are available as a Supplemental File. The base sequence in this study of Escherichia coli strain TFYM 16S rRNA encoding gene is available as a near full length sequence of this gene at GenBank: MK332445.1.

## References

[ref-1] Abd-Alla MH, Morsy FM, El-Enany A-WE (2011). Hydrogen production from rotten dates by sequential three stages fermentation. International Journal of Hydrogen Energy.

[ref-2] Alokikaa, Anu, Kumar A, Kumar V, Singh B (2021). Cellulosic and hemicellulosic fractions of sugarcane bagasse: Potential, challenges and future perspective. International Journal of Biological Macromolecules.

[ref-3] Baruah J, Nath BK, Sharma R, Kumar S, Deka RC, Baruah DC, Kalita E (2018). Recent trends in the pretreatment of lignocellulosic biomass for value-added products. Frontiers in Energy Research.

[ref-4] Bhatia SK, Jagtap SS, Bedekar AA, Bhatia RK, Rajendran K, Pugazhendhi A, Rao CV, Atabani AE, Kumar G, Yang Y-H (2021). Renewable biohydrogen production from lignocellulosic biomass using fermentation and integration of systems with other energy generation technologies. Science of the Total Environment.

[ref-5] Brenner DJ, Krieg NR, Staley JT, Garrity GM (2005). Bergey’s manual of systematic bacteriology.

[ref-6] Budiman PM, Wu TY (2018). Role of chemicals addition in affecting biohydrogen production through photofermentation. Energy Conversion and Management.

[ref-7] Camacho F, González-Tello P, Jurado E, Robles A (1996). Microcrystalline-cellulose hydrolysis with concentrated sulphuric acid. Journal of Chemical Technology & Biotechnology.

[ref-8] Cantero DA, Martinez C, Bermejo MD, Cocero MJ (2015). Simultaneous and selective recovery of cellulose and hemicellulose fractions from wheat bran by supercritical water hydrolysis. Green Chemistry.

[ref-9] Chang JK-W, Duret X, Berberi V, Zahedi-Niaki H, Lavoie J-M (2018). Two-step thermochemical cellulose hydrolysis with partial neutralization for glucose production. Frontiers in Chemistry.

[ref-10] Cheng C-L, Chang J-S (2011). Hydrolysis of lignocellulose feedstock by novel cellulases originating from Pseudomonas sp. CL3 for fermentative hydrogen production. Bioresource Technology.

[ref-11] Chong ML, Sabaratnam V, Shirai Y, Hassan MA (2009). Biohydrogen production from biomass and industrial wastes by dark fermentation. International Journal of Hydrogen Energy.

[ref-12] Chu C-Y, Wu S-Y, Tsai C-Y, Lin C-Y (2011). Kinetics of cotton cellulose hydrolysis using concentrated acid and fermentative hydrogen production from hydrolysate. International Journal of Hydrogen Energy.

[ref-13] Cuzens JC, Miller JR (1997). Acid hydrolysis of bagasse for ethanol production. Renewable Energy.

[ref-14] Dauptain K, Trably E, Santa-Catalina G, Bernet N, Carrere H (2020). Role of indigenous bacteria in dark fermentation of organic substrates. Bioresource Technology.

[ref-15] El-Zawawy WK, Ibrahim MM, Abdel-Fattah YR, Soliman NA, Mahmoud MM (2011). Acid and enzyme hydrolysis to convert pretreated lignocellulose materials into glucose for ethanol production. Carbohydrate Polymers.

[ref-16] Fan Z, Yuan L, Chatterjee R (2009). Increased hydrogen production by genetic engineering of *Escherichia coli*. PLOS ONE.

[ref-17] Felsenstein J (1985). Confidence limits on phylogenies: an approach using the bootstrap. Evolution.

[ref-18] Ghosh D, Hallenbeck PC (2009a). Fermentative hydrogen yields from different sugars by batch cultures of metabolically engineered *Escherichia coli* DJT135. International Journal of Hydrogen Energy.

[ref-19] Ghosh D, Hallenbeck PC (2009b). Response surface methodology for process parameter optimization of hydrogen yield by the metabolically engineered strain *Escherichia coli* DJT135. Bioresource Technology.

[ref-20] Hay JXW, Wu TY, Ng BJ, Juan JC, Jahim JM (2016). Reusing pulp and paper mill effluent as a bioresource to produce biohydrogen through ultrasonicated *Rhodobacter sphaeroides*. Energy Conversion and Management.

[ref-21] Heinonen J, Tamminen A, Uusitalo J, Sainio T (2012). Ethanol production from wood via concentrated acid hydrolysis, chromatographic separation, and fermentation. Journal of Chemical Technology & Biotechnology.

[ref-22] Helm RF, Huang Z, Edwards D, Leeson H, Peery W, Potts M (2000). Structural characterization of the released polysaccharide of desiccation-tolerant *Nostoc commune* DRH-1. Journal of Bacteriology.

[ref-23] Hill DR, Keenan TW, Helm RF, Potts M, Crowe LM, Crowe JH (1997). Extracellular polysaccharide of *Nostoc commune* (Cyanobacteria) inhibits fusion of membrane vesicles during desiccation. Journal of Applied Phycology.

[ref-24] Hill DR, Peat A, Potts M (1994). Biochemistry and structure of the glycan secreted by desiccation-tolerant *Nostoc commune* (cyanobacteria). Protoplasma.

[ref-25] Ho MC, Ong VZ, Wu TY (2019). Potential use of alkaline hydrogen peroxide in lignocellulosic biomass pretreatment and valorization—a review. Renewable & Sustainable Energy Reviews.

[ref-26] Iranmahboob J, Nadim F, Monemi S (2002). Optimizing acid-hydrolysis: a critical step for production of ethanol from mixed wood chips. Biomass & Bioenergy.

[ref-27] Junyapoon S, Buala W, Phunpruch S (2011). Hydrogen production with *Escherichia coli* isolated from municipal sewage sludge. Thammasat International Journal of Science and Technology.

[ref-28] Kapdan IK, Kargi F (2006). Bio-hydrogen production from waste materials *Enz*. Enzyme and Microbial Technology.

[ref-29] Kim S, Seol E, Oh YK, Wang G, Park S (2009). Hydrogen production and metabolic flux analysis of metabolically engineered *Escherichia coli* strains. International Journal of Hydrogen Energy.

[ref-30] Kumar S, Stecher G, Li M, Knyaz C, Tamura K (2018). MEGA X Molecular Evolutionary Genetics Analysis across computing platforms. Molecular Biology and Evolution.

[ref-31] Lane DJ, Stackebrandt E, Goodfellow M (1991). 16S/23S rRNA sequencing. Nucleic acid technique sin bacterial systematics.

[ref-32] Lo YC, Huang C-Y, Fu T-N, Chen C-Y, Chang J-S (2009). Fermentative hydrogen production from hydrolyzed cellulose feedstock prepared with a thermophilic anaerobic bacterial isolate. International Journal of Hydrogen Energy.

[ref-33] MacFaddin J (1985). Media for isolation-cultivation-identification-maintenance of medical bacteria.

[ref-34] Maeda T, Sanchez-Torres V, Wood T (2007). Enhanced hydrogen production from glucose by metabolically engineered *Escherichia coli*. Applied Microbiology and Biotechnology.

[ref-35] Manish S, Venkatesh KV, Banerjee R (2007). Metabolic flux analysis of biological hydrogen production by *Escherichia coli*. International Journal of Hydrogen Energy.

[ref-36] Merali Z, Collins SRA, Elliston A, Wilson DR, Käsper A, Waldron KW (2015). Characterization of cell wall components of wheat bran following hydrothermal pretreatment and fractionation. Biotechnology for Biofuels.

[ref-37] Morsy FM (2011). Hydrogen production from acid hydrolyzed molasses by the hydrogen overproducing *Escherichia coli* strain HD701 and subsequent use of the waste bacterial biomass for biosorption of Cd(II) and Zn(II). International Journal of Hydrogen Energy.

[ref-38] Morsy FM (2014). Hydrogen production by *Escherichia coli* without nitrogen sparging and subsequent use of the waste culture for fast mass scale one-pot green synthesis of silver nanoparticles. International Journal of Hydrogen Energy.

[ref-39] Morsy FM (2015). CO_2_-free biohydrogen production by mixed dark and photofermentation bacteria from sorghum starch using a modified simple purification and collection system. Energy.

[ref-40] Morsy FM (2017). Synergistic dark and photo-fermentation continuous system for hydrogen production from molasses by *Clostridium acetobutylicum* ATCC 824 and *Rhodobacter capsulatus* DSM 1710. The Journal of Photochemistry and Photobiology B: Biology.

[ref-41] Morsy FM, Elbadry M, El-Sayed WS, Abd El-Hady D (2019). Dark and photofermentation H2 production from hydrolyzed biomass of the potent extracellular polysaccharides producing cyanobacterium *Nostoc commune* and intracellular polysaccharide (glycogen) enriched *Anabaena variabilis* NIES-2095 *Int*. International Journal of Hydrogen Energy.

[ref-42] Morsy FM, Elbahloul Y, Elbadry M (2019). Photoheterotrophic growth of purple non-sulfur bacteria on Tris Acetate Phosphate Yeast extract (TAPY) medium and its hydrogen productivity in light under nitrogen deprivation. International Journal of Hydrogen Energy.

[ref-43] Morsy FM, Kuzuha S, Takani Y, Sakamoto T (2008). Novel thermostable glycosidases in the extracellular matrix of the terrestrail cyanobacterium *Nostoc commune*. The Journal of General and Applied Microbiology.

[ref-44] Muradov NZ, Veziroglu TN (2008). Green path from fossil-based to hydrogen economy: an overview of carbon-neutral technologies. International Journal of Hydrogen Energy.

[ref-45] Nath K, Das D (2004). Improvement of fermentative hydrogen production: various approaches. Applied Microbiology and Biotechnology.

[ref-46] Nelson NA (1944). photometric adaptation of the Somogyi method for the determination of glucose. Journal of Biological Chemistry.

[ref-47] Ni J, Wang H, Chen Y, She Z, Na H, Zhu J (2013). A novel facile two-step method for producing glucose from cellulose. Bioresource Technology.

[ref-48] Noblecourt A, Christophe G, Larroche C, Fontanille P (2018). Hydrogen production by dark fermentation from pre-fermented depackaging food wastes. Bioresource Technology.

[ref-49] Noparat P, Prasertsan P, Sompong O (2011). Isolation and characterization of high hydrogen-producing strain *Clostridium beijerinckii* PS-3 from fermented oil palm sap. International Journal of Hydrogen Energy.

[ref-50] Pattra S, Sangyoka S, Boonmee M, Reungsang A (2008). Bio-hydrogen production from the fermentation of sugarcane bagasse hydrolysate by *Clostridium butyricum*. International Journal of Hydrogen Energy.

[ref-51] Penfold DW, Forster CF, Macaskie LE (2003). Increased hydrogen production by *Escherichia coli* strain HD701 in comparison with the wild-type parent strain MC4100. Enzyme and Microbial Technology.

[ref-52] Pulidindi IN, Kimchi BB, Gedanken A (2014). Can cellulose be a sustainable feedstock for bioethanol production?. Renew Energy.

[ref-53] Rosales-Colunga LM, Razo-Flores E, Rodriguez ADL (2012). Fermentation of lactose and its constituent sugars by *Escherichia coli* WDHL: impact on hydrogen production. Bioresource Technology.

[ref-54] Saitou N, Nei M (1987). The neighbor-joining method: a new method for reconstructing phylogenetic trees. Molecular Biology and Evolution.

[ref-55] Sanger F, Nicklen S, Coulson AR (1977). DNA sequencing with chain-termination inhibitors. Proceedings of the National Academy of Sciences of the United States of America.

[ref-56] Sanjuán R, Anzaldo J, Vargas J, Turrado J, Patt R (2001). Morphological and chemical composition of pith and fibers from Mexican sugarcane bagasse. Holz Roh Werkstoff.

[ref-57] Scheller HV, Ulvskov P (2010). Hemicelluloses. Annual Review of Plant Biology.

[ref-58] Sınağ A, Gülbay S, Uskan B, Güllü M (2009). Comparative studies of intermediates produced from hydrothermal treatments of sawdust and cellulose. The Journal of Supercritical Fluids.

[ref-59] Sukumaran RK, Singhania RR, Pandey A (2005). Microbial cellulases Production, applications and challenges. Journal of Scientific and Industrial Research.

[ref-60] Sun Y, Cheng J (2002). Hydrolysis of lignocellulose materials for ethanol production: a review. Bioresource Technology.

[ref-61] Taherdazeh MJ, Karimi K (2007). Acid–based hydrolysis processes for ethanol from lignocellulose materials: a review. BioResources.

[ref-62] Tamaru Y, Takani Y, Yoshida T, Sakamoto T (2005). Crucial role of extracellular polysaccharides in desiccation and freezing tolerance in the terrestrial cyanobacterium *Nostoc commune*. Applied and Environmental Microbiology.

[ref-63] Tamura K, Kumar S (2002). Evolutionary distance estimation under heterogeneous substitution pattern among lineages. Molecular Biology and Evolution.

[ref-64] Thompson D, Gibson J, Plewinak F, Jeanmougin F, Higgins G (1997). The ClastalX windows interface: flexible strategies for multiple sequence alignment aided by quality analysis tools. Nucleic Acids Research.

[ref-65] Vo HT, Widyaya VT, Jae J, Kim HS, Lee H (2014). Hydrolysis of ionic cellulose to glucose. Bioresource Technology.

[ref-66] Xiang Q, Lee YY, Pettersson PO, Torget RW (2003). Heterogeneous aspects of acid hydrolysis of a-cellulose. Applied Biochemistry and Biotechnology.

[ref-67] Yokoi H, Saitsu AS, Uchida H, Hirose J, Hayashi S, Takasaki Y (2001). Microbial hydrogen production from sweet potato starch residue. Journal of Bioscience and Bioengineering.

[ref-68] Yoon S-Y, Han S-H, Shin S-J (2014). The effect of hemicelluloses and lignin on acid hydrolysis of cellulose. Energy.

[ref-69] Yoshida A, Nishimura T, Kawaguchi H, Inui M, Yukawa H (2006). Enhanced hydrogen production from glucose using *ldh*- and *frd*-inactivated *Escherichia coli* strains. Applied Microbiology and Biotechnology.

[ref-70] Yu HQ, Zhu ZH, Hu WR, Zhang HS (2002). Hydrogen production from rice winery wastewater in an upflow anaerobic reactor by using mixed anaerobic cultures. International Journal of Hydrogen Energy.

[ref-71] Zhang H, Brunsb MA, Logan BE (2006). Biological hydrogen production by *Clostridium acetobutylicum* in an unsaturated flow reactor. Water Research.

